# Clinical Characteristics and Short-Term Prognosis of Autoimmune Encephalitis: A Single-Center Cohort Study in Changsha, China

**DOI:** 10.3389/fneur.2019.00539

**Published:** 2019-05-24

**Authors:** Shuwen Deng, Ke Qiu, Hui Liu, Xiaomei Wu, Qiang Lei, Wei Lu

**Affiliations:** Department of Neurology, The Second Xiangya Hospital, Central South University, Changsha, China

**Keywords:** autoimmune encephalitis, clinical characteristics, prognosis, immunotherapy, laboratory examination

## Abstract

**Background and Purpose:** The incidence and prevalence of autoimmune encephalitis is gradually increasing. This retrospective observational study primarily aimed to analyze the clinical characteristics of autoimmune encephalitis patients in the Second Xiangya Hospital and report patient prognoses after immunotherapy.

**Methods:** The clinical data of 86 patients who were diagnosed with autoimmune encephalitis from October 2014 to September 2018 were collected, and their corresponding clinical characteristics, laboratory examination, treatment, and outcome data analyzed.

**Results:** In our study, 72 patients (83.7%) were positive for anti-NMDAR (N-methyl-D-aspartate receptor) antibody; 5 patients (6%) for anti-GABABR (γ-aminobutyric acid receptor-A); 4 patients (4.7%) for anti-LGI1 (leucine-rich, glioma inactivated 1); 3 patients (3.5%) for anti-Caspr2 (contactin-associated protein-like 2) (1 patient was positive for both anti-LGI1 and anti-Caspr2 antibodies); and 3 patients (3.5%) for onconeural antibodies. Among the 86 patients diagnosed as having autoimmune encephalitis, 50% showed acute disease onset (≤2 weeks). The most common inducing factor was fever or cold (17/86, 19.8%). The main clinical symptoms included, among others, psychiatric disturbances (82.5%), epilepsy (60.5%), autonomic dysfunction (58.1%), sleep disorders (45.3%), consciousness disorders (45.3%), and speech disorders (46.5%). No significant correlation between ICU admission rates and CSF or serum antibody scores was observed. However, CSF antibody scores of (+ + +) and (++) were associated with longer lengths of hospitalization (*p* < 0.05) and a higher CSF WBC count when compared with CSF antibody scores of (+) in patients with anti-NMDAR encephalitis (*p* < 0.05). Additionally, there was no significant correlation between mRS score difference on admission and discharge (after immunotherapy) and age, sex, and choice of immune treatment, while immune therapy taken within 15 days from onset was more inclined to be associated with an mRS score difference ≥2 after immunotherapy in patients with anti-NMDAR encephalitis (*p* = 0.006).

**Conclusions:** Autoimmune encephalitis has an acute or sub-acute onset and presents with psychotic symptoms, epilepsy, and autonomic dysfunction. The sex ratio in anti-NMDAR encephalitis was nearly balanced. Infection was a major factor inducing anti-NMDAR encephalitis, and the CSF antibody scores could be helpful in determining its prognosis since these scores showed associations with hospitalization duration and CSF WBC counts.

## Introduction

Autoimmune encephalitis (AE) is characterized by an inflammatory process of the central nervous system (CNS) and neurological disorders caused by the production of aberrant, pathogenic autoantibodies (1). Autoantibodies related to AE include those against NMDAR (N-methyl-D-aspartate receptor), GABABR (γ-aminobutyric acid receptor-B), GABAAR (γ-aminobutyric acid receptor-A), IgLON5, DPPX (dipeptidyl-peptidase-like protein-6), mGluR (metabotropic glutamate receptors), onconeural antibodies such as Amphiphysin, Hu, and GAD (glutamic acid decarboxylase), and those against proteins associated with the VGKC (voltage-gated potassium channel complex), such as anti-LGI1 (leucine-rich, glioma inactivated 1) and anti-CASPR2 (contactin-associated protein-like 2) (1, 2). AE may present with a wide variety of symptoms and with an acute or sub-acute onset. The common symptoms of AE include behavioral and psychiatric disorders, autonomic disturbances, movement disorders, and seizures (2). Awareness of AE and detection of neural autoantibodies (e.g., NMDAR autoantibodies) can improve the diagnosis rate of this condition (3). However, there is a dearth of large-sample studies regarding the pathogenesis, epidemiological and clinical characteristics, imaging features, and prognosis of AE. Studies on the clinical characteristics of AE were conducted in China (4–6), but most of them were case reports and had small sample sizes. The aim of the present retrospective study was to characterize the clinical characteristics and short-term prognosis (i.e., the outcome within the longest follow-up period ranging from 3 months to 4 years) of AE on the basis of auxiliary examination data and AE patient outcomes obtained at the Second Xiangya Hospital and provide data for future large-sample studies.

## Materials and Methods

### Patient Inclusion

This study was approved by the Ethics Committee of the Second Xiangya Hospital Affiliated to Central South University. In this retrospective observational cohort study, we enrolled all patients diagnosed with AE from 1 October 2014 to 30 September 2018 at the Second Xiangya Hospital, Central South University, Changsha, China. The patients included in the study fulfilled the criteria for AE as suggested by Graus et al. (7): (1) subacute onset (rapid progression <3 months) of memory deficits, altered mental status, or psychiatric symptoms; (2) new focal CNS findings, seizures unaccounted by a previously known seizure disorder, cerebrospinal fluid (CSF) pleocytosis, or magnetic resonance imaging (MRI) features suggestive of encephalitis; and (3) reasonable exclusion of alternative causes. Besides, only the patients who were confirmed with positive antibody tests were included in this study. Patients included in the study presented with a clinical diagnosis of “definite” AE. All the patients included were previously without any disabilities.

### Antibody Identification

The antibodies panel included anti-NMDAR, anti-GABABR, onconeural antibodies such as anti-GAD65, anti-Amphiphysin, anti-Hu and anti-Yo antibodies and those against proteins associated with the VGKC. Antibodies testing were done through indirect immunofluorescence testing (IIFT) or cell-based assays (BCA) in the Guangzhou King Med Center for Clinical Laboratory. Anti-GABAAR, anti-lgLON5, anti-DPPX, and anti-mGluR were not included in our study because of detection limitation. Following the guidelines of Guangzhou King Med Center for Clinical Laboratory, antibody scoring was defined according to the titer. The titer in CSF <1:3.2 or in serum <1:32 were scored as (+). Titers in CSF ≥1:3.2 and <1:32 or in serum ≥1:32 and <1:100 were scored as (++). Titers in CSF ≥1:32 or in serum ≥1:100 were scored as (+ + +).

### Data Collection and Analysis

Chart review was conducted to collect data regarding demographics, inducement, neurologic symptoms, neurological signs, serologic/CSF (inflammatory markers, neural autoantibody) findings, neurophysiologic (EEG), radiological (brain MRI, chest, and abdomen CT), immunotherapy, and prognosis. Disability caused by AE was evaluated at admission using the modified Rankin Scale (mRS). Clinical outcome was evaluated by mRS values at discharge from hospital and at the last follow-up. The cure rate of AE was defined as the proportion of patients with mRS of 0 at the longest follow-up after treatment. Relapse of AE was defined as new onset or worsening of symptoms, occurring after at least 2 months of improvement or stabilization (8).

### Statistics

Descriptive statistics such as median value and percentage were used to analyze the clinical data. Fisher exact tests for categorical variables were conducted to compare groups. One-way analysis of variance with Sidak-Holm *post-hoc* test was used to assess WBC count in CSF and lengths of hospitalization between samples of patients with different CSF antibodies concentrations in AE. The significance level α was set at 0.05, 2-tailed. Statistics were done using the SPSS IBM version 22 and Graphs were drawn using Graph Pad Prism 6 for MacOS.

## 3 Results

### Demographic Data and Characteristics of Patients With AE

We identified 86 patients who met the criteria for “definite” AE, including 48 men (55.8%) and 38 women (44.2%) with a median age of 32.9 years (range: 1–77 years). From October 2014 to September 2015, 14 patients were diagnosed as having AE, including 7 men (50%) and 7 women (50%) with a median age of 21.1 years. From October 2017 to September 2018, 29 patients were diagnosed as having AE, including 19 men (65.5%) and 10 women (34.5%) with a median age of 38.2 years. The proportion of cases of AE in relation to cases of CNS infection during the same period has also increased in recent years (Supplementary Figure 1). Even though the number of confirmed AE cases has continued to increase yearly, a deeper understanding of the disease and the rational use of immunotherapy have gradually improved the cure rate of AE in our hospital (Table 1).

**Table 1:** Demographic data and characteristic of the patients.

**Characteristics (years)**	**2014.10–2015.9**	**2015.10–2016.9**	**2017.10–2017.9**	**2018.10–2018.9**	**Total**
Patients	14	18	25	29	86
Age	21.1	33.9	32.6	38.2	32.9
Sex (M/F)	7:7	11:7	11:14	19:10	48:38
Cure rate (n, %)	64.3%	80%	84.3%	90%	81.3%
Death rate (n, %)	7%	5%	0	5%	6%
Relapse rate (n, %)	14.3%	5%	9.3%	5%	8%

*AE, autoimmune encephalitis; CNS, central nervous system*.

Among the 86 patients, 72 patients (83.7%) were positive for anti-NMDAR antibody; 5 patients (6%) for anti-GABABR antibody; 4 patients (4.7%) for anti-LGI1 antibody; 3 patients (3.5%) for anti-Caspr2 antibody (1 patient showed both anti-LGI1 and anti-Caspr2 antibodies); and 3 patients (3.5%) for onconeural antibodies. Different types of encephalitis showed different sex ratios and median age. There was no significant difference between the number of male and female patients with anti-NMDAR encephalitis (*p* > 0.05). The death rates, cure rates, and relapse rates in the different types of AE groups are shown in Supplementary Table 1. Seven patients relapsed during the follow-up including five cases who were positive for anti-NMDAR encephalitis (M/F: 1:4, relapse time interval range from 4 months to 1 years), one case who was positive for LGI1 encephalitis (male, relapse time interval 3 months), and one case who was positive for anti-GAD encephalitis (female, relapse time interval 6 months).

### Clinical Symptoms of the Patients

The most common inducing factor was fever or colds (17/86, 19.8%). Some uncommon inducing factors like diarrhea or psychological trauma were seen in anti-NMDAR encephalitis. Diarrhea, as the prodromal symptom, was found in patients with positive anti-NMDAR antibody with a titer of 1:3.2 in CSF; thus, the diagnosis of anti-NMDAR encephalitis was definite. However, diarrhea is also a known prodromal symptom in patients with anti-DPPX (9). However, due to the detection limitation, DPPX antibody could not be detected. Thus, it remains unknown whether anti-NMDAR antibody can co-exist with anti-DPPX antibody. Comprehensive screening tests with complete panels of known AE antigens can be done in larger cohort to assess such coexistence. Among the 86 patients, 50% showed acute-onset (≤2 weeks) disease, 33.7% showed sub-acute onset (2 weeks to 1 month) disease, and 16.3% showed chronic onset (≥1 month) disease. Different types of AE have different onset forms (Table 2).

**Table 2:** Inducement, types of onset of the patients.

**Characteristic**	**Patients**					
	**Autoimmune encephalitis**	**Anti-NMDAR encephalitis**	**LGI1 antibody encephalitis**	**GABABR antibody encephalitis**	**Caspr2 antibody encephalitis**	**Onconeural antibody associated encephalitis**
	**(*n* = 86)**	**(*n* = 72)**	**(*n* = 4)**	**(*n* = 5)**	**(*n* = 3)**	**(*n* = 3)**
**INDUCEMENT**						
Fever/influenza (n, %)	19.8%	30.5%	25%	60%	33.3%	66.7%
Diarrhea (n, %)	1.2%	1.4%	0	0	0	0
Trauma psychic (n, %)	1.2%	1.4%	0	0	0	0
**TYPES OF ONSET**						
Acute (≤2W) (n, %)	50%	51.4%	50%	60%	33.3%	0
Sub-acute (n, %)	33.7%	34.7%	0	40%	0	66.7%
Chronic (≥1M) (n, %)	16.3%	15.4%	50%	0	66.7%	33.3%

*NMDAR, N-methyl D-aspartate receptor; LGI1, leucine-rich glioma inactivated 1; GABABR, γ-aminobutyric acid B receptor; Caspr2, contactin-associated protein-like*.

**Figure 1:**
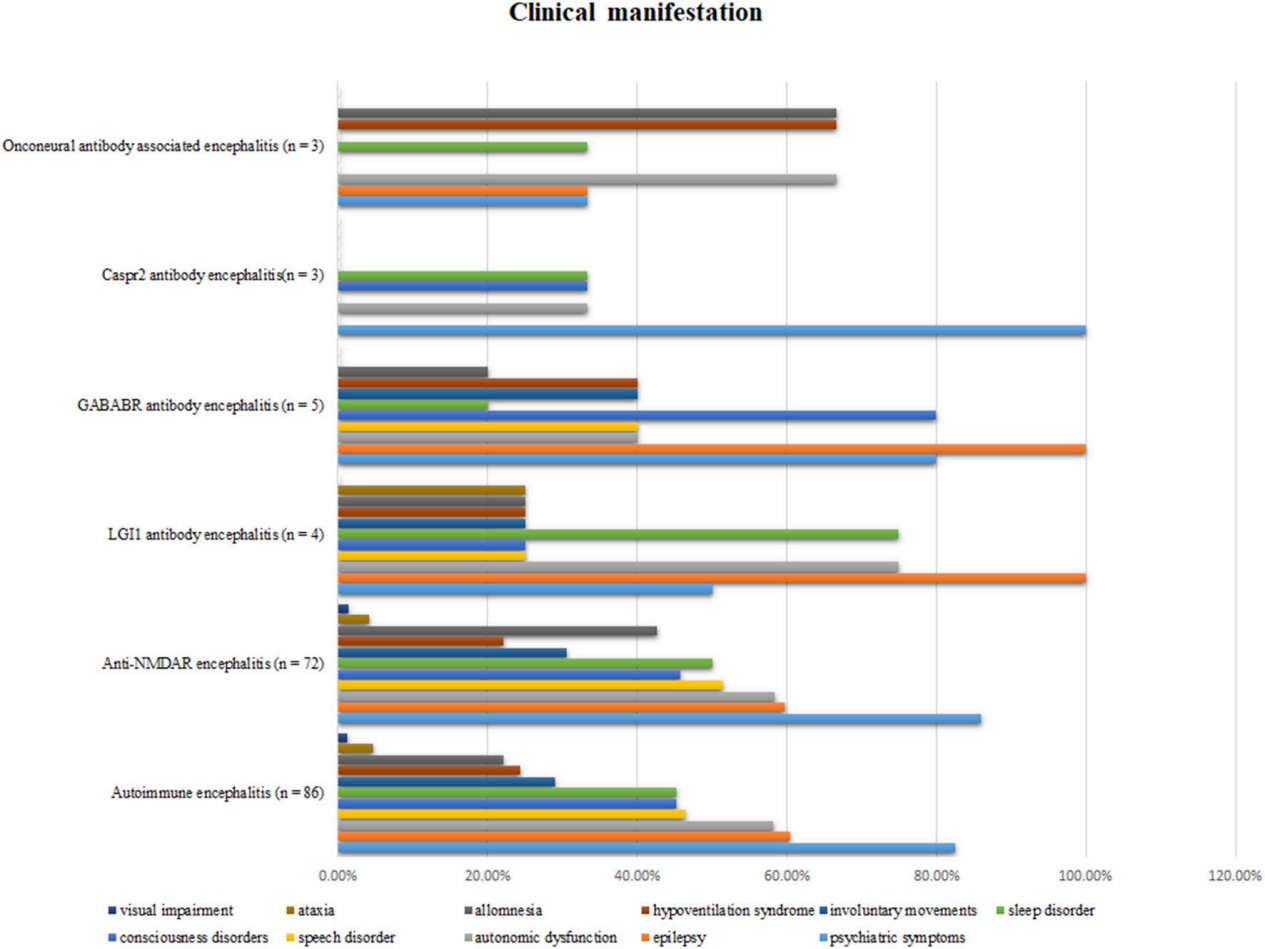
Clinical manifestations in different types of autoimmune encephalitis.

Psychiatric disturbance was the most common clinical manifestation (71 patients; 82.5%). Other common clinical manifestations included epilepsy (60.5%), autonomic dysfunction (58.1%), speech disorder (46.5%), sleep disorder (45.3%), and consciousness disorders (45.3%). The different clinical features of the five types of AE are shown in Figure 1.

### Laboratory Examination of the Patients

White blood cell (WBC) counts in blood increased in 48.3% of AE patients. Among the 86 patients, only one had an abnormal renal function (1.1%), while 13 (15.1%) patients had an abnormal liver function. For pathogen infection testing, HSV (herpes simplex virus) infection was the most common inducement in anti-NMDAR encephalitis (*n* = 9), accounting for 12.5% of the cases (Supplementary Table 2). CSF and serum for HSV PCR and IgM antibody measurements were obtained from patients during admission. HSV encephalitis was confirmed in these patients by testing CSF or serum. Subsequently, these patients had a second phase of NMDAR encephalitis about a month after discharge from the hospital.

The positive rates for thyroid-like thyroglobulin antibody (TGAB) and thyroid peroxidase antibody (TPO Ab) in different types of AE are shown in Supplementary Table 2. Meanwhile, other autoimmune antibodies such as antinuclear antibody (ANA)/extractable nuclear antigen (ENA) (*n* = 8, 9.3%) and autoimmune hepatitis (*n* = 4, 4.6%) were found in AE patients. The positive ratio of specific serum tumor markers was 2.3% (*n* = 2), while increased ferritin levels were found in six patients with AE (6.9%) (Supplementary Table 2).

### Antibodies Related to Autoimmune Antibodies

All patients underwent CSF examination, and 37 patients (43.0%) showed abnormal CSF pressure. Elevated WBC counts in the CSF were observed in 46 patients (53.4%). The maximum leukocyte level observed was 120 × 10^6^/l. Elevated total protein levels were also found in five patients (5.8%). Eight patients (9.3%) showed decreased Cl levels. Reduced total Cl levels in serum were found in one patient with anti-NMDAR encephalitis and one patient with anti-LGI1 encephalitis (Table 3). In addition, different antibody scores in the serum or CSF in the different types of AE are shown in Supplementary Table 3.

**Table 3:** CSF examination of the patients.

**CSF examination**	**Patients**					
	**Autoimmune encephalitis**	**Anti-NMDAR encephalitis**	**LGI1 antibody encephalitis**	**GABABR antibody encephalitis**	**Caspr2 antibody encephalitis**	**Onconeural antibody associated encephalitis**
	**(*n* = 86)**	**(*n* = 72)**	**(*n* = 4)**	**(*n* = 5)**	**(*n* = 3)**	**(*n* = 3)**
**CSF ANALYSIS**						
WBC, × 10^6^/L↑ (n, %)	53.4%	56.9%	0	60%	33.3%	33.3%
Protein, mg/L↑ (n, %)	5.8%	6.9%	0	0	0	0
Cl, mmol/l ↓ (n, %)	9.3%	9.7%	0	20%	0	0
Glu, mmol/l ↑ (n, %)	2.3%	2.8%	0	0	0	0
pressure↑/↓ (n, %)	43.0%	41.7%	25%	20%	66.7%	66.7%
Na, mmol/l ↓ (n, %) (Serum)	2.3%	1.4%	25%	0	0	0

*NMDAR, N-methyl D-aspartate receptor; LGI1, leucine-rich glioma inactivated 1; GABABR, γ-aminobutyric acid B receptor; Caspr2, contactin-associated protein-like 2; WBC, White Blood Cell*.

### ICU Admission Rate, Length of Hospitalization, mRS Score, and CSF WBC Counts in Patients With Different Antibody Scores for CSF or Serum

The ICU admission rates in the different types of AE are shown in Supplementary Table 3. Due to the unequal numbers of patients in the different types of AE, statistical analysis was done only for anti-NMDAR encephalitis. The results showed no significant correlation between ICU admission rates and CSF antibody scores in NMDAR encephalitis (+ vs. ++, *p* = 0.585; ++ vs.+ + +, *p* = 0.415; + vs. + + +, *p* = 0.254). Besides, there was no significant correlation between the ICU admission rate and serum antibody scores in anti-NMDAR encephalitis (+ vs. ++, *p* = 0.134; ++ vs. + + +, *p* = 0.454; + vs. + + +, *p* = 1). It is important to note that the ICU admission rate is high in GABABR encephalitis because most of these patients have status epilepticus and need sedative anesthetics under intensive care.

However, longer durations of hospitalization were found to be associated with higher CSF antibody scores in anti-NMDAR encephalitis (Supplementary Table 4). A comparison of the durations of hospitalization associated with different CSF antibody scores showed that patients with anti-NMDAR encephalitis with CSF antibody scores of (+ + +) and (++) had longer durations of hospitalization than those with CSF antibody scores of (+) (*p* < 0.05) (Supplementary Figure 2).

We also grouped the patients by mRS value ≤2 and mRS value >2 to investigate the association between mRS value on admission and different antibody scores. The proportions of patients with mRS score >2 and different CSF or serum antibody scores are presented in Supplementary Table 5. Further statistical analysis revealed that there was no significant correlation between mRS scores >2 or ≤2 before admission and different CSF antibody scores in patients with anti-NMDAR encephalitis (Supplementary Figure 3).

Finally, a comparison of the CSF WBC counts associated with different CSF or serum antibody scores showed that CSF antibody scores of (+ + +) and (++) were associated with a higher CSF WBC count in comparison with CSF antibody scores of (+) (*p* < 0.05). There was no difference between CSF antibody scores of (++) and (+ + +) (*p* > 0.05) (Supplementary Figure 4).

### Auxiliary Examinations of the Patients

**Figure 2:**
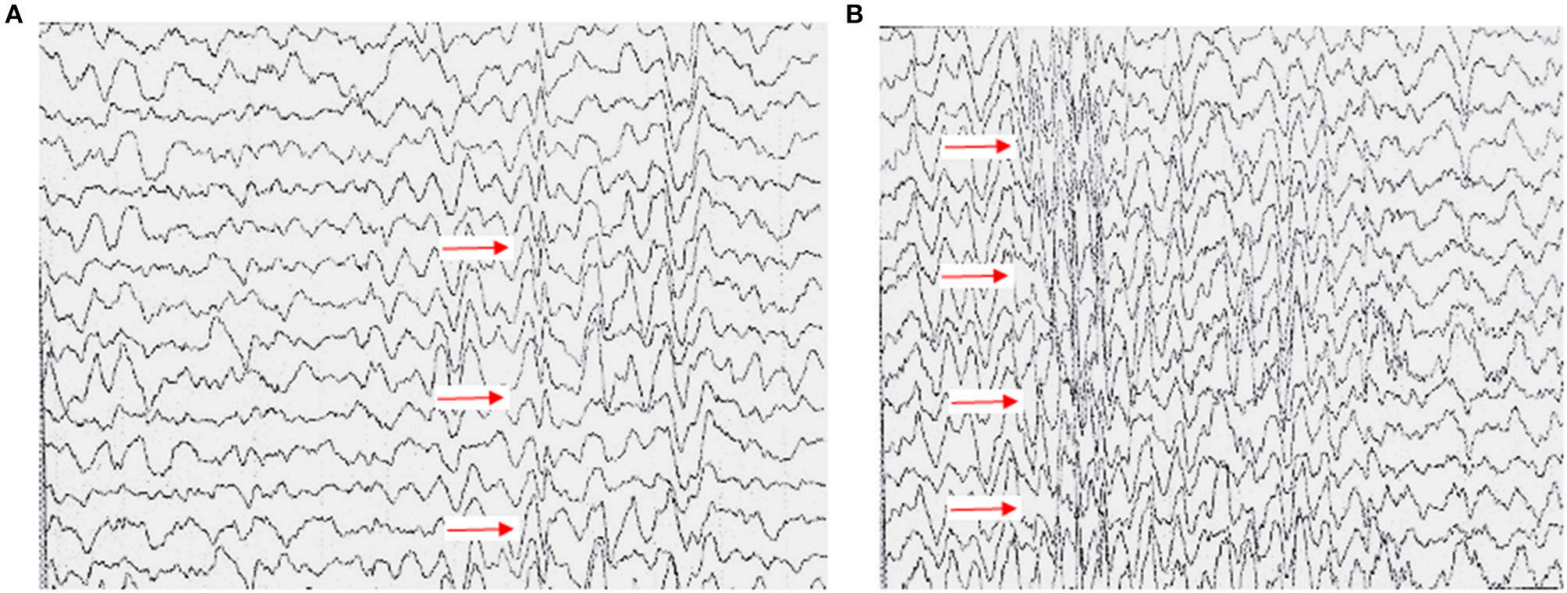
Typical EEG presentation in a case of anti-NMDAR encephalitis. **(A)** High-amplitude 2–5 c/s slow wave activity in the right cerebral hemisphere, mainly in the right central, parietal, and occipital regions (a sharp-slow complex wave can be seen). **(B)** All showed slow wave activity, with diffuse spike and wave discharges 3c/s.

EEG abnormalities were observed in 61 patients (71%); examination data were not available for 21 patients (24.4%), and normal EEG data were obtained for the remaining patients (Supplementary Table 6). The common abnormal regions of EEG were in the frontal (48.8%) and area centralis (32.6%) (Supplementary Figure 5). The proportion of slow waves in abnormal EEG was 52.3%. A typical abnormal EEG showed diffusely slow waves and sharp waves in each brain region in Figure 2.

**Figure 3:**
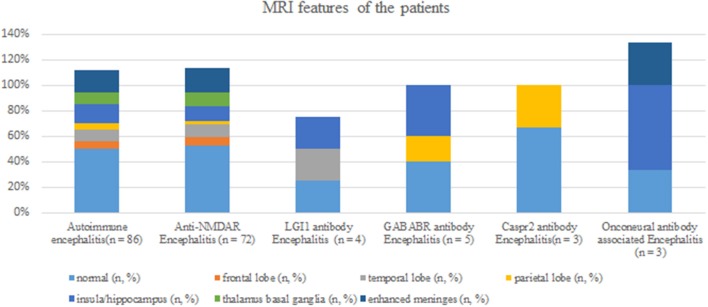
MRI features of patients with different types of autoimmune encephalitis.

**Figure 4:**
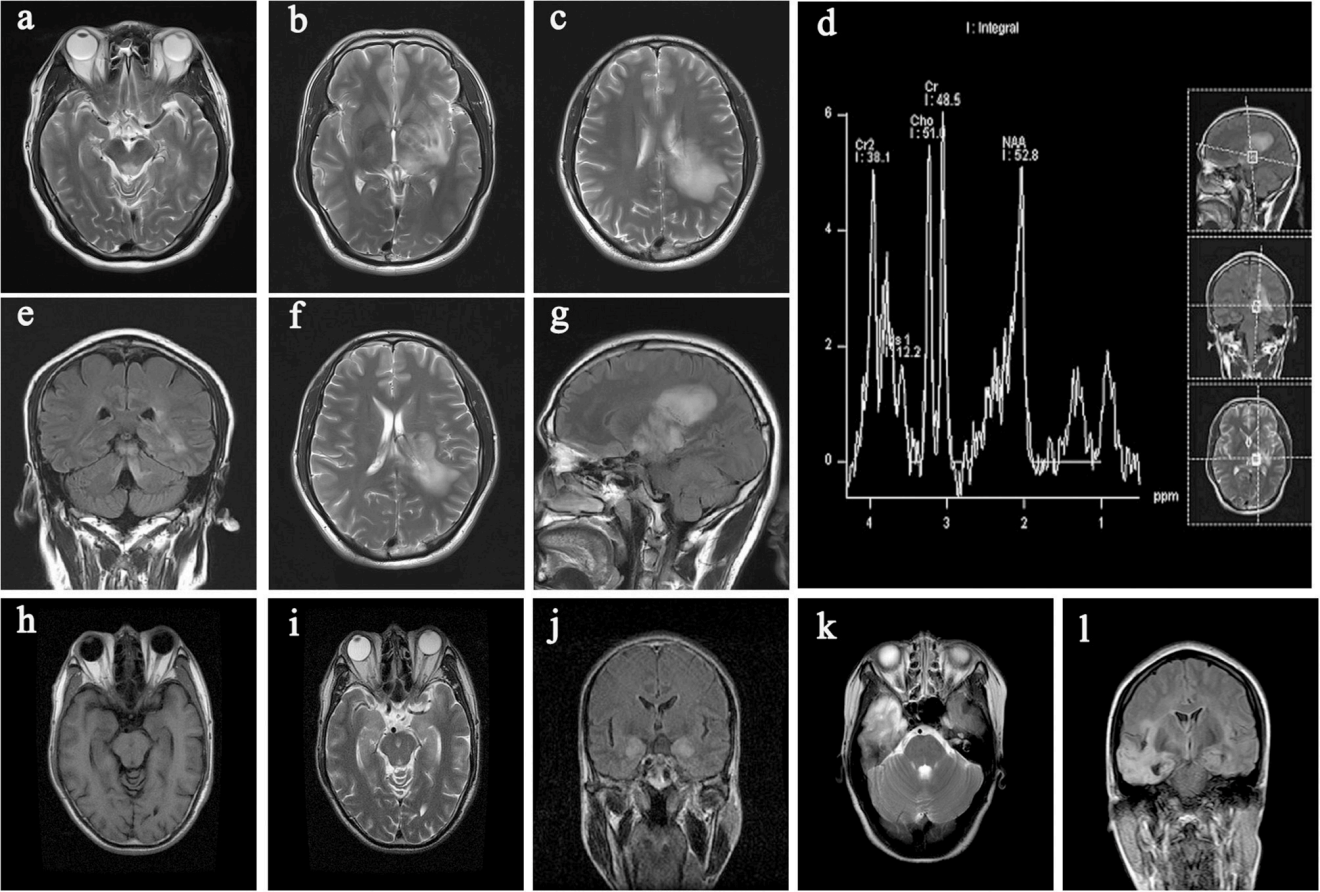
Typical imaging data from the collected cases. **(a,e)** lesion in the midbrain; **(b–d,f,g)** lesion in the cerebral peduncle, hippocampus, thalamus, and basal ganglia (left); **(h–j)** lesion in the hippocampus (bilateral); **(k,l)** lesion in the temporal lobe (right).

Brain MRI abnormalities were observed in 43 patients (50%); examination data were not available in one patient (1.2%); and normal MRI data were obtained in the remaining patients. Among the 43 patients showing brain MRI abnormalities, lesions related to AE were mainly found in the insula/hippocampus (15.1%) (Figure 3). Some typical foci observed in MRI are presented in Figure 4.

Lung CT scan abnormalities were observed in 48 patients (55.8%); examination data were not available in eight patients (9.3%); and normal lung CT scan data were obtained in the remaining patients. Among the 48 patients, 2 (2.3%) showed lung cancer and 25 (29.1%) showed pulmonary inflammation. Two patients diagnosed with lung cancer were found to have CSF positive for anti-GABABR antibody and anti-NMDAR antibody. Abdominal CT or ultrasound abnormalities were observed in 54 patients (62.8%); examination data were not available in 10 patients (11.6%); and normal abdominal CT or ultrasound data were obtained in the remaining patients. Among the 54 patients, 2 (2.3%) showed teratomas and both were identified as having anti-NMDAR encephalitis (Supplementary Table 6).

### Different Predictor Variables and Curative Effect in Patients

Based on the mRS score differences on admission and discharge (after immunotherapy), the patients were divided into two groups with mRS score difference ≥2 and mRS score difference < 2. Therapy included only the use of steroid or in combination with plasma exchange, gamma-globulin, or immunosuppressive agents like cyclophosphamide. Steroid treatment was administered as a high-dose intravenous methylprednisolone pulse therapy which was followed by tapering to an oral dose and the patients were discharged and maintained on this oral dose until recovery. The association between mRS score differences on admission and discharge (after immunotherapy) and different predictor variables, including age, sex, choice of immune treatment, and time to immune therapy was compared in anti-NMDAR encephalitis. The results revealed no significant correlation between mRS score difference and age (*p* = 0.254), sex (*p* = 0.533), and choice of immune treatment (*p* = 0.805). However, immune therapy administered within 15 days from onset was associated with a higher rate of mRS score difference ≥2 (*p* = 0.006) (Table 4).

**Table 4:** Different predictor's variables and curative effect in patients.

**Different predictors variables**	**mRS difference after Immunotherapy (≥2)**				
	**Anti-NMDAR encephalitis**	**LGI1 antibody encephalitis**	**GABABR antibody encephalitis**	**Caspr2 antibody encephalitis**	**Onconeural antibody associated encephalitis**
	**(*n* = 69)**	**(*n* = 4)**	**(*n* = 4)**	**(*n* = 3)**	**(*n* = 3)**
**AGE**					
Elder (≥45 years)	8/9(88.9%)	1/3(33.3%)	2/3(66.7%)	0/1(0%)	1/1(100%)
Youth (<45 years)	39/60 (65%)	1/1(100%)	0/1(0%)	2/2(100%)	1/2(50%)
**SEX**					
Male	24/37(64.9%)	1/3(33.3%)	2/3(66.7%)	1/1(100%)	1/1(100%)
Female	23/32(71.9%)	1/1(100%)	0/1(0%)	1/2(50%)	1/2(50%)
**IMMUNE TREATMENT**					
Only steroids	22/33(66.7%)	2/4(50%)	1/1(100%)	2/2(100%)	1/1(100%)
Two or more Immunotherapies	25/36(69.4%)	–	1/3(33.3%)	0/1(0%)	1/2(50%)
**TIME TO IMMUNE THERAPY, d**					
<15	28/46 (60.9%)	3/4(75%)	4/4(100%)	2/3(66.7%)	2/2(100%)
≥15	6/23(26.1%)	–	–	–	1/1(100%)

*NMDAR, N-methyl D-aspartate receptor; LGI1, leucine-rich glioma inactivated 1; GABABR, γ-aminobutyric acid B receptor; Caspr2, contactin-associated protein-like 2; mRS, modified Rankin Scale; IVIG, Intravenous Immune Globulin. Exclusion of patients who did not receive immunotherapy (n = 82)*.

## Discussion

### Clinical Features of Anti-NMDAR Encephalitis

Among the AE cases diagnosed at our hospital, anti-NMDA encephalitis accounts for the largest proportion. Previous data revealed that Anti-NMDAR encephalitis occurred in 20–40-year-old young women (10). However, in our study, the sex ratio for anti-NMDAR encephalitis was nearly balanced. This is likely due to ethnic differences and the insufficient sample size in our study. Ovarian teratoma is the most frequent tumor found in anti-NMDAR encephalitis (11), although only two patients with ovarian teratoma, out of the 72 patients with anti-NMDAR encephalitis, were seen in our study. Similarly to our study, HSV has been shown by other studies to be an important inducer of anti-NMDAR antibody encephalitis (12). To date, researchers have speculated that viral triggers create a pro-inflammatory state that “primes” the immune system, including CNS-resident immune cells termed microglia, to become overactive and create an autoimmune response against the CNS (13). Besides HSV, some patients also showed positive findings for HIV, TP, rubella virus, *Mycoplasma pneumoniae*, Epstein-Barr virus, and cytomegalovirus. Among the antigens mentioned above, HSV (14), HIV (15), Epstein-Barr virus (16), and *Mycoplasma pneumoniae* (17), which were reported in a previous study, can be inducing factors of AE. Haneche et al. reported a case of anti-NMDAR encephalitis in an HIV-infected woman, and they stated that the HIV-1 glycoprotein gp120 can decrease the NR1 subunit in NMDA receptor and might participate in the dysfunction of NMDA receptors (18).

The mode of onset in anti-NMDAR encephalitis is always acute or sub-acute. The common symptoms in anti-NMDAR encephalitis have been reported to include psychiatric symptoms, epilepsy, involuntary movement, and sleep disorders, and our result is similar to a previous study (19). Besides, autonomic dysfunction, including dysrhythmia, hyperhidrosis, alternating bradycardia/tachycardia, hypotension/hypertension and hypothermia/hyperthermia, are easily neglected or misdiagnosed although they can threaten patient survival and are worth noting (20). There are still some rare clinical manifestations, such as visual impairment and ataxia, which we found in anti-NMDAR encephalitis patients in our study. A similar case involving a 67-year-old man presenting with encephalopathy and psychosis, impaired visual fixation, and ataxia was reported, and the CSF of that patient contained anti-Hu, anti-CRMP-5, and anti-NMDAR autoantibodies (21). However, the appearance of visual impairment or ataxia needs to be taken seriously, because AE can coexist with demyelinating diseases and may occur in some individuals sequentially or simultaneously. Therefore, these patients were tested for specific autoantibodies (such as anti-AQP4 and anti-MOG antibodies) and underwent genetic screening, yielding negative results.

The main auxiliary examinations significant for the diagnosis of anti-NMDAR encephalitis included EEG, MRI, and CSF examinations. However, to the best of our knowledge, the present study is the first to highlight the CSF antibody scores as significantly associated with length of hospitalization and CSF WBC counts, both of which can affect clinical prognosis (22). The levels of CSF antibodies are more closely related to the clinical outcome when compared with the levels of serum antibodies (23).

### Clinical Features of GABABR, LGI1, and CASPR2 Encephalitis

The clinical features of the other types of AE are similar to those described in previous studies. GABABR encephalitis shows a homogenous distribution in both male and female populations with a median age of 60 years compared with the median age of 49.4 years in our study (11). Bronchial carcinoma and neuroendocrine tumors were the most frequent tumors found in GABABR encephalitis (11). Seizures are the most common clinical presentation in GABABR encephalitis and always accompanied by altered consciousness. Autonomic dysfunction can induce acute mortality which also should be taken seriously (24).

Positive anti-CASPR2 or anti-LGI1 antibody mainly occurred in male patients with a median age of 60 years compared with a median age of 40–50 years in our study (11). Marked clinical overlaps between patients with either anti-LGI1 or anti-CASPR2 antibodies include frequent focal seizures, prominent amnesia, dysautonomia, neuromyotonia, and neuropathic pain (25). Because fewer cases were admitted, the above characteristics were not confirmed in this study. However, the difference in clinical features between patients with either anti-LGI1 or anti-CASPR2 antibodies were confirmed in our study. For example, seizures and hyponatremia were more common in anti-LGI1-IgG positive patients while neuropathic pain was more common in anti-CASPR2-IgG positive patients (26).

### Therapy

Steroid is the first choice for AE and shows a good curative effect in most patients. The most commonly used combined immunotherapeutic agent is intravenous steroids and immunoglobulin (IVIG). Steroids can also be combined with plasma exchange. In some cases, plasma exchange can be used in patients with slow recovery treated with corticosteroids and intravenous immunoglobulin (IVIG) (22). If there is little or no clinical improvement after the treatments mention above, second-line therapy including rituximab or cyclophosphamide should be implemented (27). A good outcome is depended on early recognition and timely treatment (11). With the longest follow-up period, most patients in our study showed favorable prognoses and relapses are, fortunately, uncommon in AE.

### The Concurrent of AE and Other Immune Diseases

Some researchers believe that AE represents a manifestation of neuronal autoimmunity within a pathogenic spectrum of disorders that includes thyroid gastric autoimmune disorders, paraneoplastic syndromes, stiff-man syndrome, and neuromuscular autoimmunity (28). Therefore, attention should be given to the detection of other autoimmune diseases once the diagnosis of AE has been made.

Autoantibodies including ANA/ENA, anti-mitochondrial antibody (AMA-M2), TPO, and TGAB, were found to coexist with anti-NMDAR antibody in our study. Arinuma et al. reported that the anti-dsDNA antibodies may cross-react with the NMDA receptor in SLE patients and presents with diffuse neuropsychiatric manifestations; however, the antibodies were determined with only a single method (29). Besides, Guan et al. demonstrated that patients with positive anti-thyroid antibodies showed trends toward better disease outcomes than patients with negative anti-thyroid antibodies, and these antibodies might suggest a propensity to autoimmunity that may play an important role in non-tumor-associated anti-NMDA receptor encephalitis (30). However, using larger cohorts, it will be helpful to assess whether the autoantibodies mentioned above can cross-react with NMDA receptors or not.

Notably, during the follow-up period, two patients (one male and one female) were readmitted to our department due to a sharp visual decline with concomitant anti-MOG and anti-NMDA antibodies in their CSF, although the symptoms of AE had disappeared after the last treatment. Similar findings were reported in several cases (31–33), where it was named overlapping MOG-ab disease and NMDAR encephalitis (MNOS) by the authors. Since oligodendrocytes do contain NMDAR, it is reasonable to speculate that the immune attack targeting myelin may involve NMDAR simultaneously (33). Researchers suggest that in patients with MOG-ab disease who exhibit psychiatric behaviors or cognitive dysfunction and supratentorial lesions, the co-existence of NMDAR encephalitis should be considered and anti-NMDA receptor antibody expression should be evaluated because they found that 100% of patients with MNOS had supratentorial lesions (31).

### Limitations and Strengths

Limitations of our study include its retrospective methodology. In addition, patients were not screened comprehensively for the complete known panel of AE antigens, including anti-AMPA-R, anti-GABAAR, and anti-glycine-R antibodies; therefore, the extrapolation of this study is limited. However, this study is one of the largest series of autoimmune encephalitis patients in China. It was conducted in a regional central hospital which has certain representativeness and provides a basis for future study.

## Conclusions

AE primarily presents in the form of mental disorders, epilepsy, and involuntary movement, and is often accompanied by sleep disorders. In addition to immunological assessment of cerebrospinal fluid and serum, EEG and head MRI can provide important diagnostic basis for AE diagnosis. Screening for lung, ovarian, colorectal, and prostate cancer, in addition to others, is indispensable. Once AE is diagnosed, early immune-modulatory treatment can alleviate the severity of the disease and improve the cure rate.

## Data Availability

No datasets were generated or analyzed for this study.

## Author Contributions

WL designed the study. SD, KQ, and HL performed the data collection and statistical analysis. SD performed the literature search and wrote the paper. XW, QL, and WL reviewed and edited the manuscript. WL takes responsibility for the integrity of the work from its inception to publishing. All authors read and approved the manuscript.

### Conflict of Interest Statement

The authors declare that the research was conducted in the absence of any commercial or financial relationships that could be construed as a potential conflict of interest.
