# Supplementary material for: Clinical Characteristics and Short-Term Prognosis of Autoimmune Encephalitis: A Single-Center Cohort Study in Changsha, China
Source: Front Neurol. 2019 May 24;10:539. doi: 10.3389/fneur.2019.00539 (PMC6543891; doi:10.3389/fneur.2019.00539)
Supplement: Supplementary file 1 [file Data_Sheet_1.docx]

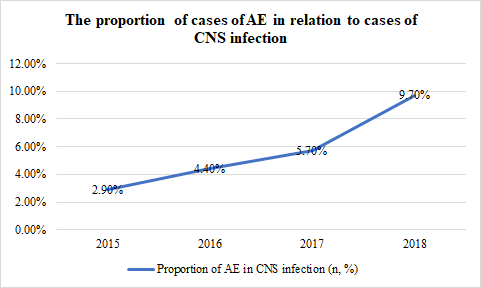


**Supplementary Figure 1:** The proportion of cases of AE in relation to cases of CNS infection.

**Abbreviations:** CNS= central nervous system; AE= autoimmune encephalitis


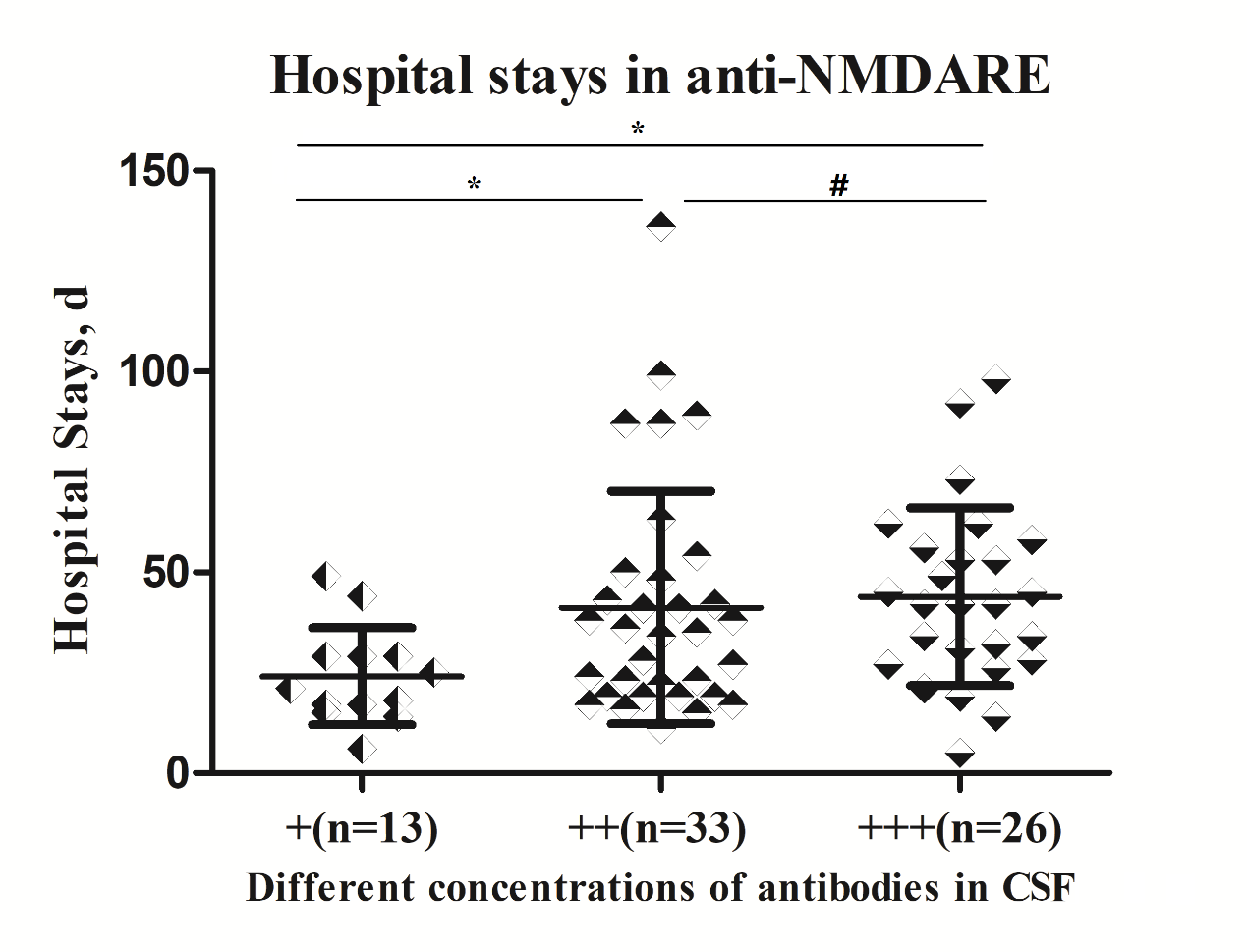


**Supplementary Figure 2**: Length of hospitalization in the different CSF antibodies concentrations groups. (^*^ p＜0.05; ^#^ p＞0.05).

**Abbreviations:**CSF= cerebrospinal fluid;anti-NMDARE=anti-N-methyl D-aspartate receptor encephalitis


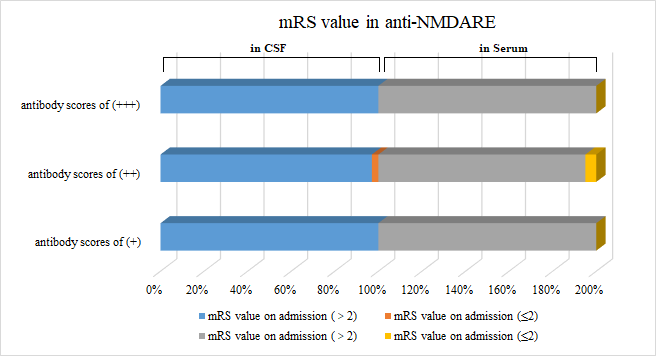


**Supplementary Figure 3**:mRS value in different antibodies concentrations groups in anti-NMDARE.

**Abbreviations:**CSF= cerebrospinal fluid;anti-NMDARE=anti-N-methyl D-aspartate receptor encephalitis; mRS= modified Rankin Scale


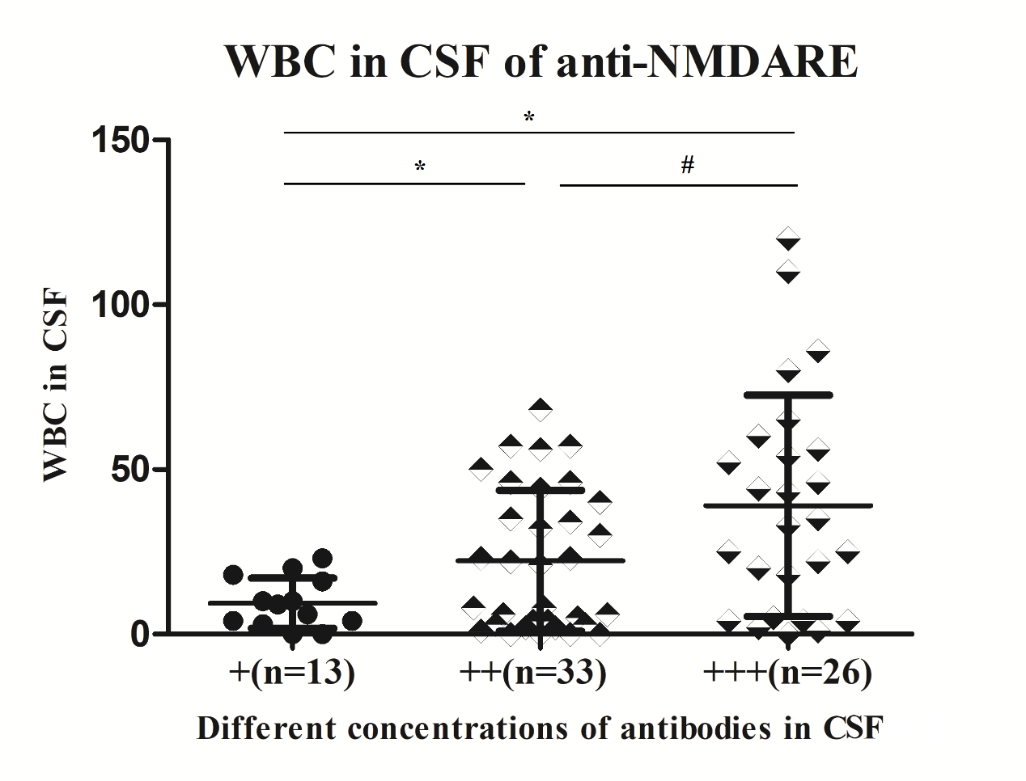


**Supplementary Figure 4**: The count of WBC in the different CSF antibodies concentrations groups. (*p＜0.05; ^#^p＞0.05)

**Abbreviations:**CSF=cerebrospinal fluid;anti-NMDARE=anti-N-methyl D-aspartate receptor encephalitis; WBC= white blood cell


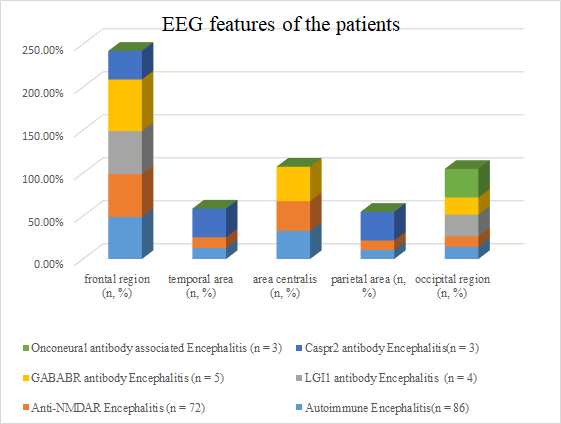


**Supplementary Figure 5**: EEG features of patients with different types of autoimmune encephalitis.

**Abbreviations:** NMDAR=N-methyl D-aspartate receptor; LGI1=leucine-rich glioma inactivated 1; GABABR=γ-aminobutyric acid B receptor; Caspr2=contactin-associated protein-like 2
