# Supplementary material for: Clinical Characteristics and Short-Term Prognosis of Autoimmune Encephalitis: A Single-Center Cohort Study in Changsha, China
Source: Front Neurol. 2019 May 24;10:539. doi: 10.3389/fneur.2019.00539 (PMC6543891; doi:10.3389/fneur.2019.00539)
Supplement: Supplementary file 2 [file Data_Sheet_2.docx]

| Demographic data | Patients | | | | | | | | | | |
| --- | --- | --- | --- | --- | --- | --- | --- | --- | --- | --- | --- |
|  | **Autoimmune encephalitis**  **(n = 86)** | **Anti-NMDAR**  **encephalitis**  **(n = 72)** | | **LGI1 antibody**  **encephalitis**  **(n = 4)** | | **GABABR antibody encephalitis**  **(n = 5)** | | **Caspr2 antibody encephalitis**  **(n = 3)** | | **Onconeural antibody associated encephalitis**  **(n = 3)** | |
| Sex (M/F) | 48:38 | | 40:32 | | 3:1 | | 3:2 | | 1:2 | | 1:2 |
| Age | 32.9 | | 30.4 | | 49.7 | | 49.4 | | 41.3 | | 34.3 |
| Cure rate (n, %) | 81.3% | | 79.2% | | 100% | | 80% | | 100% | | 66.7% |
| Death rate (n, %) | 6% | | 5.6% | | 0 | | 20% | | 0 | | 0 |
| Relapse rate (n, %) | 8% | | 6.9% | | 25% | | 0 | | 0 | | 33.3% |

**Supplementary Table 1**

**Demographic data and characteristic in different types of AE**

**Abbreviations:** NMDAR=N-methyl D-aspartate receptor; LGI1=leucine-rich glioma inactivated 1; GABABR=γ-aminobutyric acid B receptor; Caspr2=contactin-associated protein-like 2

**Supplementary Table 2**

**Laboratory examination of the patients**

| Laboratory  Examination | Patients | | | | | | | |
| --- | --- | --- | --- | --- | --- | --- | --- | --- |
|  | **Autoimmune encephalitis**  **(n = 86)** | **Anti-NMDAR**  **encephalitis**  **(n = 72)** | **LGI1 antibody**  **encephalitis**  **(n = 4)** | **GABABR antibody encephalitis**  **(n = 5)** | | **Caspr2 antibody encephalitis**  **(n = 3)** | **Onconeural antibody associated encephalitis**  **(n = 3)** | |
| Organic function evaluation |  | | | | | | | |
| AST/ALT, ×100↑ (n, %)  BUN/Cr↑ (n, %)  WBC/N % ↑ (n, %) | 15.1% | 13.9% | 25% | 0 | | 33.3% | 33.3% | |
|  | 1.2% | 1.4% | 0 | 0 | | 0 | 0 | |
|  | 48.3% | 43.1% | 50% | 60% | | 33.3% | 0 | |
| Contagion test |  | | | | | | | |
| viral hepatitis (+) (n, %)  HIV (+) (n, %)  TP (+) (n, %)  HSV (+) (n, %)  Other virus infections (+) (n, %) | 4.6% | 5.6% | 0 | 0 | | 0 | 33.3% | |
|  | 1.2% | 1.4% | 0 | 0 | | 0 | 0 | |
|  | 1.2%  10.5%  5.8% | 1.4%  12.5%  5.6% | 0  0  0 | 0  0  0 | | 0  0  0 | 0  0  33.3% | |
| Serum autoantibody  and tumor markers |  | | | | | | | |
| TGAB (+) (n, %)  TPO (+) (n, %)  ENA/ANA (+) (n, %)  Autoimmune hepatitis (+) (n, %)  Ferritin↑ (n, %)  Tumor markers (n, %) | 10.4% | 6.9% | 50% | | 0 | 33.3% | | 66.7% |
|  | 14% | 12.5% | 50% | | 0 | 33.3% | | 33.3% |
|  | 9.3% | 8.3% | 50% | | 0 | 33.3% | | 0 |
|  | 4.6% | 4.1% | 50% | | 0 | 0 | | 0 |
|  | 6.9% | 4.2% | 0 | | 0 | 33.3% | | 33.3% |
|  | 2.3% | 2.8% | 0 | | 0 | 0 | | 0 |

**Abbreviations:** NMDAR=N-methyl D-aspartate receptor; LGI1=leucine-rich glioma inactivated 1; GABABR=γ-aminobutyric acid B receptor; Caspr2=contactin-associated protein-like 2; ANA = antinuclear; ALT= Alanine aminotransferase; AST=Aspartate aminotransferase; HIV: Human Immune Deficiency Virus; HSV= Herpes Simplex virus; TP= Treponema; ENA= Extractable nuclear antigen; TGAB=thyroglobulin antibody; TPO= Thyroid peroxidase

**Supplementary Table 3**

**The ICU admission rate in different types of AE**

| Concentration of antibodies | ICU admission | | | | | |
| --- | --- | --- | --- | --- | --- | --- |
|  | **Autoimmune encephalitis**  **(n = 86)** | **Anti-NMDAR**  **encephalitis**  **(n = 72)** | **LGI1 antibody**  **encephalitis**  **(n = 4)** | **GABABR antibody encephalitis**  **(n = 5)** | **Caspr2 antibody encephalitis**  **(n = 3)** | Onconeural antibody associated encephalitis  (n = 3) |
| Antibodies (CSF) |  | | | | | |
| Total | 34/79(43.0%) | 30/72(41.7%) | 0/2(0%) | 4/5(80%) | - | - |
| +  ++  +++ | 4/14 (28.6%) | 4/13(30.8%) | - | 0/1(0%) | - | - |
|  | 15/37(40.5%) | 13/33(39.4%) | 0/2(0%) | 2/2(100%) | - | - |
|  | 15/28(53.6%) | 13/26(50%) | - | 2/2(100%) | - | - |
| Antibodies (Serum) |  | | | | | |
| Total | 17/61(41.0%) | 22/46(47.8%) | 0/4(0%) | 4/5(80%) | 0/3(0%) | 2/3(66.7%) |
| +  ++  +++ | 13/24(54.2%) | 11/18(61%) | 0/1(0%) | 1/2(50%) | 0/2(0%) | 1/1(100%) |
|  | 9/27(33.3%) | 7/20(35%) | 0/3(0%) | 2/2(100%) | - | 1/2(50%) |
|  | 5/10(50%) | 4/8(50%) | - | 1/1(100%) | 0/1(0%) | - |

**Abbreviations:** NMDAR=N-methyl D-aspartate receptor; LGI1=leucine-rich glioma inactivated 1; GABABR=γ-aminobutyric acid B receptor; Caspr2=contactin-associated protein-like 2; CSF= cerebrospinal fluid

**Supplementary Table 4**

**The median** **hospital stays in different types of AE**

| Concentration of antibodies | Median hospital stays (Days) | | | | | |
| --- | --- | --- | --- | --- | --- | --- |
|  | **Autoimmune encephalitis**  **(n = 86)** | **Anti-NMDAR**  **encephalitis**  **(n = 72)** | **LGI1 antibody**  **encephalitis**  **(n = 4)** | **GABABR antibody encephalitis**  **(n = 5)** | **C****aspr2 antibody encephalitis**  **(n = 3)** | Onconeural antibody associated encephalitis  (n = 3) |
| Antibodies (CSF) |  | | | | | |
| +  ++  +++ | 25.5 | 24.1 | - | 44 | - | - |
|  | 38.5 | 41.2 | 19.5 | 12.5 | - | - |
|  | 44.5 | 43.9 | - | 52 | - | - |
| Antibodies (Serum) |  | | | | | |
| +  ++  +++ | 40.2 | 40.9 | 37 | 27.5 | 26.5 | 20 |
|  | 40.4 | 43.2 | 22.3 | 24 | - | 39.5 |
|  | 42.5 | 39.8 | - | 70 | 37 | - |

**Abbreviations:** NMDAR=N-methyl D-aspartate receptor; LGI1=leucine-rich glioma inactivated 1; GABABR=γ-aminobutyric acid B receptor; Caspr2=contactin-associated protein-like 2; CSF= cerebrospinal fluid

**Supplementary Table 5.**

**mRS score on admission in different antibody scoring**

| Concentration of antibodies | mRS value on admission (＞2) | | | | | |
| --- | --- | --- | --- | --- | --- | --- |
|  | **Autoimmune encephalitis**  **(n = 86)** | **Anti-NMDAR**  **encephalitis**  **(n = 72)** | **LGI1 antibody**  **encephalitis**  **(n = 4)** | **GABABR antibody encephalitis**  **(n = 5)** | **Caspr2 antibody encephalitis**  **(n = 3)** | Onconeural antibody associated encephalitis (n = 3) |
| Antibodies (CSF) |  | | | | | |
| +  ++  +++ | 14/14 (100%) | 13/13(100%) | - | 1/1(100%) | - | - |
|  | 35/37(94.6%) | 32/33(97.0%) | 2/2(100%) | 1/2(50%) | - | - |
|  | 28/28(100%) | 26/26(100%) | - | 2/2(100%) | - | - |
| Antibodies (Serum) |  | | | | | |
| +  ++  +++ | 24/24 (100%) | 18/18(100%) | 1/1(100%) | 2/2(100%) | 2/2(100%) | 1/1(100%) |
|  | 24/27 (88.9%) | 19/20(95.0%) | 2/3(66.7%) | 1/2(50%) | - | 2/2(100%) |
|  | 10/10 (100%) | 8/8(100%) | - | 1/1(100%) | 1/1(100%) | - |

**Abbreviations:** mRS= modified Rankin Scale; NMDAR=N-methyl D-aspartate receptor; LGI1=leucine-rich glioma inactivated 1; GABABR=γ-aminobutyric acid B receptor; Caspr2=contactin-associated protein-like 2; CSF= cerebrospinal fluid

**Supplementary Table 6.**

**Auxiliary examinations of the patients**

| Auxiliary examinations | Patients | | | | | |
| --- | --- | --- | --- | --- | --- | --- |
|  | **Autoimmune encephalitis**  **(n = 86)** | **Anti-NMDAR**  **encephalitis**  **(n = 72)** | **LGI1 antibody**  **encephalitis**  **(n = 4)** | **GABABR antibody encephalitis**  **(n = 5)** | **Caspr2 antibody encephalitis**  **(n = 3)** | **Onconeural antibody associated encephalitis**  **(n = 3)** |
| EEG |  | | | | | |
| normal  mildly abnormal (n, %)  moderate abnormal (n, %)  highly abnormal (n, %)  NA | 4.6% | 5.6% | 0 | 0 | 0 | 0 |
|  | 23.3% | 23.6% | 0 | 40% | 0 | 33.3% |
|  | 33.7% | 31.9% | 75% | 40% | 0 | 33.3% |
|  | 14% | 15.3% | 0 | 0 | 33.3% | 0 |
|  | 24.4% | 23.6% | 25% | 20% | 66.7% | 33.3% |
| slow wave (n, %)  Sharp wave (n, %) | 52.3% | 52.8% | 75% | 40% | 33.3% | 33.3% |
|  | 4.7% | 5.6% | 0 | 0 | 0 | 0 |
| Lung CT Scan |  |  |  |  |  |  |
| Normal/NA  tumor  inflammatory | 44.2% | 47.2% | 25% | 0 | 0 | 66.7% |
|  | 2.3% | 1.4% | 0 | 20% | 0 | 0 |
|  | 29.1% | 29.2% | 0 | 60% | 0 | 33.3% |
| Abdominal CT /ultrasound |  |  |  |  |  |  |
| Normal/NA  tumor | 37.2% | 37.5% | 50% | 20% | 33.3% | 66.7% |
|  | 2.3% | 2.8% | 0 | 0 | 0 | 0 |

**Abbreviations:** NMDAR=N-methyl D-aspartate receptor; LGI1=leucine-rich glioma inactivated 1; GABABR=γ-aminobutyric acid B receptor; Caspr2=contactin-associated protein-like 2; EEG= electroencephalograph
